# Development of a novel biomarker model for predicting preoperative lymph node metastatic extent in esophageal squamous cell carcinoma^1^

**DOI:** 10.18632/oncotarget.22399

**Published:** 2017-11-11

**Authors:** Zhao Ma, Xianxian Wu, Bo Xu, Hongjing Jiang, Peng Tang, Jie Yue, Mingquan Ma, Chuangui Chen, Hongdian Zhang, Zhentao Yu

**Affiliations:** ^1^ Department of Esophageal Cancer, Tianjin Medical University Cancer Institute and Hospital, Key Laboratory of Prevention and Therapy of Tianjin, Tianjin's Clinical Research Center for Cancer, National Clinical Research Center of Cancer, Tianjin, China; ^2^ Department of Molecular Radiation Oncology, Tianjin Medical University Cancer Institute and Hospital, Tianjin, China

**Keywords:** esophageal squamous cell cancer, lymph node metastasis, biomarker, risk prediction, squamous cell carcinoma antigen

## Abstract

The number and range of lymph node metastasis (LNM) are critical prognostic factors in esophageal squamous cell carcinoma (ESCC). Preoperative serum biomarkers are reported to be associated with LNM. However, whether these markers can precisely predict the extent of LNM is not known. The aim of this study was to evaluate the predictive value of preoperative serum SCC-Ag, Cyfra21-1, CEA, CA19-9 and CA72-4 for LNM number and range by retrospectively investigating 577 ESCC patients undergone esophagectomy from 2007-2010. In this study, the positive rate of SCC-Ag and CA19-9 were associated with pN stage. Significant differences were found in CEA and CA19-9 between pN0-1 stage patients and pN2-3 stage patients. However, in subgroup analysis (patients with pN0-1), significant difference was found only in SCC-Ag between pN0 and pN1 stage patients (*P*=0.003). Middle thoracic ESCC patients were Chosen to analyze the correlation between the range of LNM and biomarkers. SCC-Ag was correlated with paraesophageal and paracardial lymph nodes, but not correlated with subcarinal and left gastric artery lymph nodes. Interestingly, the results of CEA were opposite to that of SCC-Ag. CA19-9 was associated with subcarinal and paracardial LNM (*P*=0.000, *P*=0.038). Based on the results, a model incorporated SCC-Ag, CEA and CA19-9 was constructed. The rate of patients with pN2-3 stage was 15.4% and 54.4% in group 1 and 4 of our model.

In summary, SCC-Ag was associated with early lymph node metastatic stage, and CEA and CA19-9 have a close relationship with advanced lymph node metastatic stage. The model combining SCC-Ag, CEA and CA19-9 might help identify the preoperative extent of LNM for a subgroup of ESCC patients that can be benefited from neoadjuvant therapy.

## INTRODUCTION

The prognosis of esophageal squamous cell carcinoma (ESCC) depends on the clinical stage of the primary tumor and the extent of lymph node metastasis (LNM) [1]. Lymph node status could be classified by LNM number or stations based on the latest version of the UICC/AJCC TNM classification (^8^th edition)[2] or Japanese Classification of Esophageal Cancer (^11^th edition)[3]. These two LNM classification methods define the extent of LNM by analyzing the metastatic number and range.

Serum biomarkers are biologic or biochemical substances that are produced by tumor cells, and are commonly used for identifying cancer, indicating treatment outcomes and predicting prognosis. In ESCC, it has been reported that preoperative biomarkers have a close relationship with tumor burden, including tumor invasion, LNM and the survival rate [4, 5]. But there has been no studies focusing on the correlation between biomarkers and metastatic extent of lymph node which was more valuable for clinicians to evaluate operative indication.

Squamous cell carcinoma antigen (SCC-Ag), carcinoembryonic antigen (CEA), cytokeratin 19 fragments (Cyfra21-1), carbohydrate antigen 19-9 (CA199) and carbohydrate antigen 72-4 (CA724) are commonly used in the management of esophageal cancer patients. SCC-Ag and Cyfra21-1 are sensitive biomarkers in malignant disease, particularly in squamous cell carcinoma [6, 7]. CEA, an oncofetal glycoprotein, is a representative biomarker that has been known to be elevated in almost all solid tumors, especially colorectal cancer [8]. In esophageal carcinoma, CEA has been reported as a diagnostic and prognostic marker [9, 10]. CA199 and CA724 are elevated in a variety of cancers, especially gastrointestinal cancer [11–13].

In the present study, (1) we clarify which preoperative serum levels of the biomarkers were associated with LNM status. (2) SCC-Ag, Cyrfra21-1, CEA and CA19-9, which showed a significant correlation with LNM, were selected to evaluate the relationship with lymph node metastatic number and stations. (3) We analyzed the diagnostic values of the model incorporated preoperative serum SCC-Ag, CEA and CA19-9 for lymph node metastatic extent in ESCC.

## RESULTS

### Patients and disease characteristics

In 577 ESCC patients, the median age of the patients was 61 years (range, 27 to 82 years). The median and interquartile range of SCC-Ag, Cyfra21-1, CEA, CA19-9 and CA72-4 were 0.7 ug/L (0.4-1.3 ug/L), 2.36 ug/L(1.69-3.26 ug/L), 2.19 ug/L(1.49- 3.11 ug/L), 8.66 U/ml (5.06-15.92 U/ml) and 1.30 U/ml (0.92-2.44 U/ml). Baseline patient disease characteristics are shown in Table 1.

**Table 1:** Clinical and biological characteristics of 577 patients in ESCC

Characteristics	No. of patients (%)(N=577)
Age, years	
≤60	274 (47.5%)
> 60	303 (52.5%)
Sex	
Male	476 (82.5%)
Female	101 (17.5%)
Tumor location	
Upper	45 (7.8%)
Middle	396 (68.6%)
Lower	136 (23.6%)
Pathological T status	
T1	21 (3.6%)
T2	105 (18.2%)
T3	235 (40.7%)
T4a	157 (27.2%)
T4b	59 (10.2%)
Pathological N status	
N0	310 (53.7%)
N1	167 (28.9%)
N2	73 (12.7%)
N3	27 (4.7%)
Tumor Grade	
Grade 1	21 (3.6%)
Grade 2	468 (81.1%)
Grade 3	88 (15.3%)
TNM stage	
I	21 (3.7%)
IIA	44 (7.6%)
IIB	180 (31.2%)
IIIA	139 (24.1%)
IIIB	33 (5.7%)
IIIC	160 (27.7%)
SCC-Ag	
Positive (> 1.5 ug/L)	102 (17.7%)
Negative (≤1.5 ug/L)	463 (80.2%)
Cyfra21-1	
Positive (> 3.3 ug/L)	140 (24.3%)
Negative (≤3.3 ug/L)	437 (75.7%)
CEA	
Positive (> 5 ug/L)	37 (6.4%)
Negative (≤5 ug/L)	540 (93.6%)
CA19-9	
Positive (>39 U/ml)	23 (4.0%)
Negative (≤39 U/ml)	554 (96.0%)
CA72-4	
Positive (> 6 U/ml)	46 (8.0%)
Negative (≤6 U/ml)	531 (92.0%)

### The correlations between the preoperative serum biomarkers and LNM

The positive rate of serum SCC-Ag, Cyfra21-1, CEA and CA19-9 were associated with LNM in the Chi-square test (Table 2; all *P*<0.05). Same statistically significant results were found when assessing the relation between the biomarkers level and LNM using the Mann-Whitney-U test (SCC-Ag, *P*=0.000; Cyfra21-1, *P*=0.049; CEA, *P*=0.032; CA19-9, *P*= 0.024; CA72-2, *P*=0.361). On the multivariate analysis, SCC-Ag and CA19-9 were independent risk factors for LNM (Table 2; *P*<0.05).

**Table 2:** Univariate and multivariate logistic regression analysis for correlation between clinical and biological characteristics and lymph node metastasis in 577 cases

Biomarkers	Lymph node metastasis		Univariate analysis		Multivariate analysis	
	Negative	Positive	χ^2^	*P* value	OR	*P* value
Age, years						
≤60	140	134	1.267	0.260	NA	NA
> 60	169	134				
Sex						
Male	247	229	3.020	0.082	NA	NA
Female	62	39				
Tumor location						
Upper	25	20	0.347	0.841	NA	NA
Middle	214	182				
Lower	70	66				
Pathological T status						
T1	19	2	22.590	**0.000**	-	0.106
T2	66	39				
T3	124	111				
T4a	70	87				
T4b	30	29				
Tumor Grade						
Grade 1	17	4	10.810	**0.004**		
Grade 2	254	214			3.881	**0.019**
Grade 3	38	50			6.096	**0.003**
Tumor length						
≤ 3cm	94	43	16.382	**0.000**	2.198	**0.000**
> 3cm	215	225				
SCC-Ag	263	201	10.138	**0.001**	1.917	**0.005**
Negative	40	62				
Positive						
Cyfra21-1			4.566	**0.033**	-	0.116
Negative	245	192				
Positive	64	76				
CEA			3.925	**0.048**	-	0.241
Negative	295	245				
Positive	14	23				
CA19-9			7.265	**0.007**	3.612	**0.010**
Negative	303	251				
Positive	6	17				
CA72-4			2.040	0.153	NA	NA
Negative	289	242				
Positive	20	26				

NA, not available (not included in multivariate analysis).

### Correlations between preoperative serum biomarkers and LNM number

SCC-Ag, Cyfra21-1, CEA and CA19-9 were chosen to analyze the correlations with pN stage. The positive rate of serum SCC-Ag and CA19-9 were associated with pN stage (Table 3; *P*=0.010 and *P*=0.004). To further examine the relationship between SCC-Ag, Cyfra21-1, CEA and CA19-9 and the LNM extent, patients with pN0 and pN1were selected (477 patients in total). In this subpopulation, the positive rate of SCC-Ag was much higher in pN1 stage patients compared to those in pN0 stage patients (23.9% vs. 13.2%, *P*=0.003), but no correlations were found in Cyfra21-1, CEA and CA19-9 (all *P*>0.05). (Table 4).

**Table 3:** Correlation between biomarkers and pN stage in 577 ESCC patients

pN stage	SCC-Ag		*P* value	Cyfra21-1		*P* value	CEA		*P* value	CA19-9		*P* value
	Negative	Positive		Negative	Positive		Negative	Positive		Negative	Positive	
N0	263	40	0.010	246	64	0.065	296	14	0.137	306	6	0.004
N1	124	39		122	45		155	12		161	6	
N2	57	15		53	20		67	8		66	7	
N3	19	8		16	11		24	3		23	4	

**Table 4:** Correlation between biomarkers and early stage lymph node metastasis in 477 pN0-1 stage patients

pN stage	SCC-Ag		*P* value	Cyfra21-1		*P* value	CEA		*P* value	CA19-9		*P* value
	Negative	Positive		Negative	Positive		Negative	Positive		Negative	Positive	
N0	263	40	0.003	246	64	0.118	296	14	0.221	304	6	0.270
N1	124	39		122	45		155	12		161	6	

For analyzing the advanced stage of LNM, patients were divided into two subgroups, pN0-1 and pN2-3. Patients with pN0-1stage were considered earlier and less extensive LNM, while patients with pN2-3 stage were considered more extensive LNM. High positive rate of preoperative serum CEA and CA199 were associated with more extensive LNM (*P*=0.039, *P*=0.000, respectively). However, no correlation was found in SCC-Ag (P=0.105) (Table 5).

**Table 5:** Correlation between biomarkers and advanced stage lymph node metastasis in 577 ESCC patients

Tumor markers	N0-1(LNM≤2)	N2-3(LNM>2)	χ^2^	*P* value
SCC-Ag				
Negative	387	76	2.176	0.140
Positive	79	23		
Cyfra21-1			2.987	0.084
Negative	368	69		
Positive	109	31		
CEA			4.242	**0.039**
Negative	451	89		
Positive	26	11		
CA19-9			15.548	**0.000**
Negative	465	89		
Positive	12	11		

### Correlations between preoperative serum biomarkers and LNM groups

Japanese Classification of Esophageal Cancer (11th edition) [3] graded pN stage(JES) based on the LNM groups which were defined according to the location of the tumor. For middle thoracic ESCC, middle thoracic paraesophageal lymph nodes and paracardial lymph nodes were involved in pN1 (JES), subcarinal lymph nodes and left gastric artery lymph nodes were involved into pN2 (JES). pN2 (JES) stage was more extensive lymphatic invasion than pN1 (JES) stage.

In middle thoracic ESCC, the positive rate of SCC-Ag was correlated with paraesophageal and paracardial lymph nodes in pN1 (JES) (all *P*<0.05), but not correlated with subcarinal and left gastric artery lymph nodes in pN2 (JES). Interestingly, the association between the positive rate of CEA and LNM groups were opposite to that in SCC-Ag. Significant results of CEA were found in pN2 group (JES) (all *P*<0.05) and not in pN1 group (JES). The positive rate of serum CA19-9 was associated with subcarinal and paracardial LNM (*P*=0.000, *P*=0.038) (Table 6).

**Table 6:** Correlation between biomarkers and lymph node metastatic groups in 396 middle thoracic ESCC

Tumor marker	SCC-Ag		*P* value	CEA		*P* value	CA19-9		*P* value
	Negative	Positive		Negative	Positive		Negative	Positive	
middle thoracic paraesophageal lymph nodes			**0.012**			0.963			0.559
Negative	267	51		301	23		311	13	
Positive	49	20		67	5		68	4	
paracardial lymph nodes									
Negative	284	52	**0.000**	322	23	0.415	333	12	**0.038**
Positive	32	19		46	5		46	5	
subcarinal lymph nodes									
Negative	281	59	0.174	327	21	**0.030**	338	10	**0.000**
Positive	35	12		41	7		41	7	
left gastric artery lymph nodes									
Negative	265	53	0.067	307	19	**0.037**	314	12	0.195
Positive	51	18		61	9		65	5	

### A biomarkers diagnostic model and risk groups to LNM

To better identify ESCC patients at high risk for LNM, we proposed a new diagnostic model by combining SCC-Ag, CEA and CA19-9, and stratified patients into four groups. Grade 1 group: negative SCC-Ag, CEA and CA19-9; Grade 2 group: positive SCC-Ag, negative CEA and negative CA19-9; Grade 3 group: negative SCC-Ag with positive CEA or positive CA19-9 or both; Grade 4 group: positive SCC-Ag with positive CEA or positive CA19-9 or both. In the patients reported here, there are 422, 91, 41 and 11 patients in the grade 1, 2, 3 and 4 groups, respectively. With this model, the analysis showed the rate of patients with pN2-3 stage in ESCC was 15.4% in grade 1 group. The rate of LNM patients was 81.8% and the rate of patients with pN2 -3 stage was 54.5% in grade 4 group. ROC curve analyses were performed, the area under the ROC curve (AUC) in predicting LNM with this model was 0.567 (*P*=0.006). The AUC in predicting LNM extent with this model was 0.563 (*P*=0.048) (Supplementary Figure 1) (Table 7).

**Table 7:** The discriminatory value of diagnostic model for lymph node metastatic extent in 577 ESCC patients

Diagnostic model	Lymph node metastasis		χ^2^	*P* value	Lymph node metastasis extent		χ^2^	*P* value
	Negative	Postitive			N0-1	N2-3		
Grade 1 group	244(57.8%)	178(42.2%)	15.240	**0.002**	357(84.6%)	65(15.4%)	14.287	**0.003**
Grade 2 group	38(41.8%)	53(58.2%)			74 (81.3%)	17(18.7%)		
Grade 3 group	18(43.9%)	23(56.1%)			30(73.2%)	11(26.8%)		
Grade 4 group	2(18.2%)	9 (81.8%)			5 (45.5%)	6(54.5%)		

## DISCUSSION

To our knowledge, our report is the largest published retrospective study to date that analyzes the correlation between preoperative serum biomarkers (SCC-Ag, Cyfra21-1, CEA, CA19-9 and CA72-4) and LNM in ESCC. More importantly, there have been no studies investigating the predictive values of these preoperative serum biomarkers to the extent of LNM. The present study found that the serum SCC-Ag, CEA and CA19-9 were closely associated with lymph node metastatic extent. Different serum biomarkers revealed different stages of LNM. The model we built will help predict LNM and further define the precise status of pN stage (N0-1 or N2-3).

ESCC has a high prevalence in Asia and easily metastasizes to lymph nodes. Neoadjuvant therapy, especially preoperative chemoradiation therapy, could downstage the tumor, significantly reduce the 3-year mortality and locoregional recurrence compared with surgery alone [15–17]. The clinical value of preoperative lymph node status identification is to decide whether patients should undergo neoadjuvant therapy first. Diagnostic imaging techniques, including computed tomography and endosonography, have limitation that cannot predict lymph node status sufficiently. Preoperative serum biomarkers, which are measured routinely prior to treatment, could provide information of LNM based on our study presented here.

SCC-Ag and Cyfra21-1 are sensitive biomarker in malignant disease, particularly in squamous carcinoma [6, 7]. In ESCC, the positive rate of SCC-Ag and Cyfra21-1 previously reported were 25.2% - 38% [4, 18–20] and 17.7%- 47.8% [5, 20]. In our study, the positive rate of SCC-Ag and Cyfra21-1 were 18.1% and 24.3%. Different positive rates of these biomarkers might result from different proportion of TNM stage and detected method. Previous studies have indicated that SCC-Ag was correlated with LNM in head and neck, cervical, penile and anal canal cancer [21–24]. In esophageal cancer, except LNM, elevated SCC-Ag was also associated with tumor size, depth of tumor invasion, TNM stage and poor survival rate [4, 5, 25]. Similar results were observed in this study and SCC-Ag was an independent risk factor for LNM. We also examined SCC-Ag in pN0-1 stage patients (LNM number ≤ 2) which could analyze early stage lymph node metastatic status. In this subgroup, only SCC-Ag was associated with LNM (*P*=0.003) and its specificity was 86.8%. Although Cyfra21-1, CEA and CA19-9 were associated with LNM in ESCC, they do not serve as precise predictors for early stage LNM.

There is a controversy for categorizing pathological N stage based on UICC/AJCC TNM classification or Japanese Classification of Esophageal Cancer. These two methods represent different aspects of LNM extent. Larger number or range of LNM stands for more extensive metastasis and later stage. Among the 4 biomarkers, only high positive rate of CEA and CA19-9 were demonstrated to associate with advanced stage LNM. There was no significant difference for SCC-Ag (*P*=0.140). These results showed SCC-Ag would be elevated at early stage of LNM and could predict early LNM status. On the contrary, CEA and CA19-9 could predict advanced LNM status.

It should be noted that our purpose to analyze the correlation between lymph node stations and biomarkers was not to predict the specific lymph node station metastatic status with markers. In Japanese Classification of Esophageal Cancer, some specific lymph node stations stand for more extensive metastasis. To confirm our thoughts above, we chose middle thoracic esophageal carcinoma patients and analyzed the status of middle thoracic paraesophageal lymph nodes (pN1, JES) and paracardial lymph nodes (pN1, JES), subcarinal lymph nodes (pN2, JES), and left gastric artery lymph nodes (pN2, JES). Interestingly, SCC-Ag was significantly associated with pN1 (JES) stage LNM (*P*<0.05), but not pN2 (JES) stage (*P*>0.05). Opposite relationships with lymph node stations were found in CEA. Similar results of CA19-9 were verified in the thoracic lymph node stations. In abdominal lymph node stations, CA19-9 was correlated with paracardial LNM. A probable reason for this discrepancy may be explained as follows. Wang et al. [26]argued that most patients had abdominal LNM accompanied by thoracic (mediastinal) LNM at the same time and the 5-year survival rate was lower than that of patients with thoracic LNM. CA19-9 could not indentify well in abdominal LNM whose lymph metastasis extent was more severe compared with thoracic LNM.

The positive rate of CEA in ESCC ranges from 9.1% to 23% [4, 18, 27]. CEA has been reported to own the function of an adhesion molecule. CEA- containing tumor cells could preferentially go into metastatic cascade [28, 29]. Kosugi et al.[4] reported that CEA had no correlation with LNM, but with distant metastasis in ESCC. However, another study revealed significant differences were found between CEA and LNM in ESCC [18]. CA19-9 is also called sialylated Lewis Antigen which is speculated to play roles in the extravasation of cancer cells from blood and the promotion of metastatic spread to distant organs [30]. CA19-9 was widely used in pancreatic cancer, colon cancer, gastric cancer, and other gastrointestinal tumors [11, 12, 31–33]. In a meta-analysis of gastric cancer, there were the correlation between elevated CA19-9 and LNM, T stage, TNM stage, vessel invasion and poor survival rate [34]. In ESCC, Zhao et al. [31] first reported CA19-9 with a new cutoff value was associated with LNM and hematogenic metastasis. Our study reveals that elevated CEA and CA19-9 are associated with advanced stage of LNM.

Together with previous findings, our study provided a LNM predictive model, which could help identify the extent of LNM. Patients with advanced stage LNM, which was classified into III stage or above (based on UICC/AJCC TNM classification), are recommended to undergo preoperative chemoradiotherapy or chemotherapy first.

Several limitations to our study exist. Since the analyses were performed retrospectively on a single institutional database, selection biases might be underestimated. Additional validation of the models using different data sets might further prove the clinical value of the model.

In conclusion, this study demonstrates the correlation between LNM and the positive rate of preoperative serum SCC-Ag, Cyfra21-1, CEA and CA19-9. SCC-Ag was associated with early lymph node metastatic stage. CEA and CA19-9 have a close relationship with advanced lymph node metastatic stage. The model incorporated SCC-Ag, CEA and CA19-9 could further increase the predictive value for the LNM status of esophageal squamous cell carcinoma.

## MATERIALS AND METHODS

### Patients and eligibility criteria

Among patients with esophageal squamous cell carcinoma undergone esophagectomy in Cancer Institute and Hospital of Tianjin Medical University from January 2007 to December 2010, the study was conducted on 577 patients whose preoperative serum SCC-Ag, Cyfra21-1, CEA, CA19-9 and CA72-4 were examined. The cases exclude from the current study based on the following criteria: (1) patients who had history of malignant disease; (2) patients who had received preoperative treatment (chemotherapy and/or radiotherapy); (3) patients who had other malignant tumors except esophageal squamous carcinoma.

The serum Cyfra21-1, CEA, CA19-9 and CA72-4 were detected within 7 days before surgery with electrochemiluminescence immunoassay (Roche Elecsys E170, Germany). The SCC-Ag was detected by chemical luminescence immunoassay (Abbott Architect i2000, America). The normal upper limit was 1.5 ug/L, 3.3 ug/L, 5 ug/L, 39 U/ml and 6 U/ml for SCC-Ag, Cyfra21-1, CEA, CA19-9 and CA72-4 respectively. The esophagectomy with 2- to 3- field lymph node dissection was taken for standard treatment. The depth of primary tumor, degree of lymph node (pN stage) and TNM staging were defined according to UICC/AJCC TNM classification (7th edition)[14]. For analyzing the relationship between lymph node metastatic range and biomarkers, the LNM grading method of Japanese Classification of Esophageal Cancer (11th edition) [3] also were used and defined as “pN stage (JES)”. JES is the abbreviation for Japanese Esophageal Society. In present study, we defined pN1 and pN2-3 (UICC/AJCC TNM classification or Japanese Classification of Esophageal Cancer) as early stage and advanced stage LNM to analyze the correlation between biomarkers and LNM extent.

### Statistical analysis

The statistical analyses were performed with the SPSS 21.0 software (ver. 21 SPSS Inc., Chicago, IL). The correlations between serum level of biomarkers and clinicopathological characteristics were assessed using the Chi-square or Mann-Whitney-U test. Significant risk factors identified by univariate analysis were further assessed by multivariate analysis using logistic regression. A receiver operating characteristic (ROC) curve was constructed. *P* < 0.05 was considered statistically significant.

## SUPPLEMENTARY MATERIALS FIGURE



## Abbreviations

(ESCC): Esophageal squamous cell carcinoma
(LNM): Lymph node metastasis
(JES): Japanese Esophageal Society
(ROC): A receiver operating characteristic
(AUC): The area under the ROC curve
