# Supplementary material for: Development of a novel biomarker model for predicting preoperative lymph node metastatic extent in esophageal squamous cell carcinoma1
Source: Oncotarget. 2017 Nov 11;8(62):105790–9. doi: 10.18632/oncotarget.22399 (PMC5739679; doi:10.18632/oncotarget.22399)
Supplement: Supplementary file 1 [file oncotarget-08-105790-s001.pdf]

Development of a novel biomarker model for predicting preoperative lymph node metastatic extent in esophageal squamous cell carcinoma[1]

SUPPLEMENTARY MATERIALS

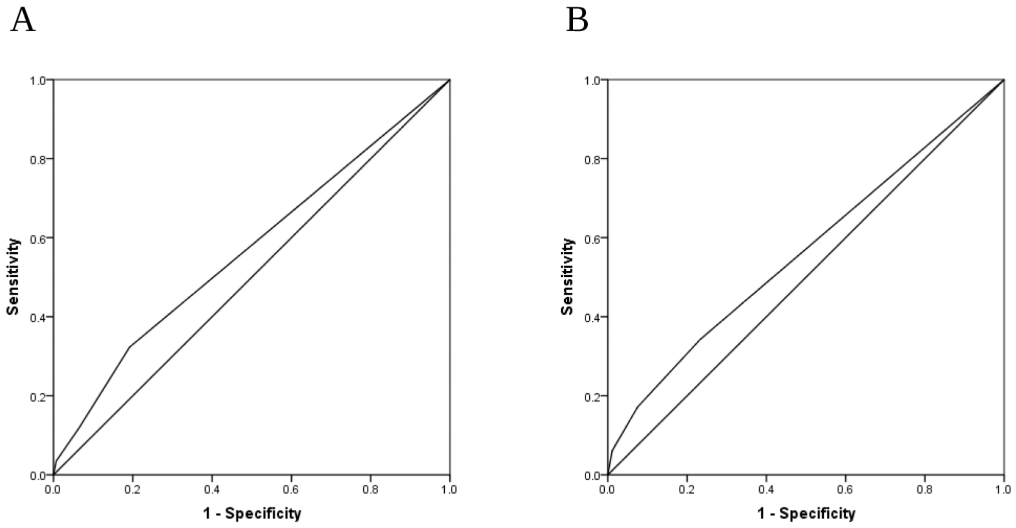

Supplementary Figure 1: Receiver operating characteristic curves for the model in predicting LNM and LNM extent. **(A)**: Model in predicting LNM; **(B)**: Model in predicting LNM extent.
